# Powerful Exact Unconditional Tests for Agreement between Two Raters with Binary Endpoints

**DOI:** 10.1371/journal.pone.0097386

**Published:** 2014-05-16

**Authors:** Guogen Shan, Gregory E. Wilding

**Affiliations:** 1 Department of Environmental and Occupational Health, Epidemiology and Biostatistics Program, School of Community Health Sciences, University of Nevada Las Vegas, Las Vegas, Nevada, United States of America; 2 Department of Biostatistics, University at Buffalo, Buffalo, New York, United States of America; University of East Piedmont, Italy

## Abstract

Asymptotic and exact conditional approaches have often been used for testing agreement between two raters with binary outcomes. The exact conditional approach is guaranteed to respect the test size as compared to the traditionally used asymptotic approach based on the standardized Cohen's kappa coefficient. An alternative to the conditional approach is an unconditional strategy which relaxes the restriction of fixed marginal totals as in the conditional approach. Three exact unconditional hypothesis testing procedures are considered in this article: an approach based on maximization, an approach based on the conditional p-value and maximization, and an approach based on estimation and maximization. We compared these testing procedures based on the commonly used Cohen's kappa with regards to test size and power. We recommend the following two exact approaches for use in practice due to power advantages: the approach based on conditional p-value and maximization and the approach based on estimation and maximization.

## Introduction

Assignment of a binary rating for a fixed number of subjects from two independent raters is often seen in scientific studies. The data from such studies can be organized in a 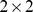 table, and it is often of interest in performing inferences regarding the agreement between raters. For example, Smedmark et al. [1] considered a study of assessment of passive inter-vertebral motion of the cervical spine. Patients from a private clinic in Stockholm were examined by two physical therapists (referred to as clinicians A and B) with similar clinical experience. Each patient was determined to have spinal stiffness or not by each rater through use of a medical testing procedure. The exam result for rotation to the right of C1–2 [1] was recorded for each patient by the raters, and the associated data is shown in Table 1. As seen in the table, both clinicians agreed that there was no stiffness for 50 patients, and spinal stiffness was present in 2 patients. There was one patient that clinician A diagnosed as having stiffness while the clinician B did not. Conversely, for the remaining 7 patients, clinician B concluded spinal stiffness was present where clinician A did not. To quantify agreement, an obvious and straightforward measurement is the probability of agreement, defined by the total number of ratings for which both raters agree, divided by the total number of patients in the study. In this example, the probability of agreement would be 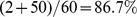.

**Table 1:** 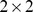
 contingency table for the agreement test.

		Clinician B		Total
		Yes	No	
Clinician A	Yes	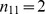	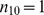	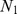
	No	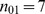	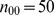	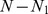
Total		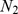	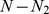	N = 60

Cohen's kappa [2], [3] is a measure of agreement adjusted for chance. It has been reported that the lower bound of the Cohen's kappa depends on the marginal totals. When the the marginal totals are very unbalanced, the delta model developed by Martin Andres and Femia Marzo [4] may be used as an alternative to the kappa. The Cohen's kappa has some desirable properties [5]. A kappa of 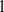 implies perfect agreement, while a kappa of less than 0 means that less agreement was observed than would be expected by chance. In the case of kappa equal to 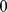, the level of agreement is seen by chance. The kappa for the previous example is 0.2793, which is considered to signify fair strength of agreement according the definition by Landis and Koch [6]. The asymptotic one-sided or two-sided p-values can be computed from the limiting distribution of the standardized kappa test statistic, a standard normal distribution [3]. In addition to the asymptotic p-value, many software programs, such as SAS PROC FREQ, also provide an exact conditional p-value due to the conditional approach [7] for testing kappa = 0. Nuisance parameters (two marginal probabilities) are accommodated in the exact conditional approach due to Fisher [7] by conditioning on marginal totals (referred to as the C approach). Given both marginal totals, the value of 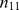 in Table 1 determines the other three counts 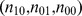. Thus, the null reference distribution is constructed by enumerating all possible 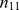. Although the type I error rate of the study is well controlled by the C approach, it may be conservative due to the small size of the sample space, especially in the small to medium sample size settings.

A number of exact unconditional procedures have been proposed [8], [9] to reduce the conservativeness of the C approach. One of them as described by Basu [9] who considers the exact unconditional approach by maximizing the tail probability over the nuisance parameter space (referred to as the M approach). This is a general approach which has been utilized for testing the equality of two independent proportions. With only the total sample size fixed, the null reference distribution for unconditional approaches produces a much larger sample space than that of the C approach. Boschloo [10] proposed another unconditional approach by combining the C approach and the M approach (referred to as the C+M approach), where the p-value from the C approach is used as a test statistic when maximizing across the nuisance parameter space. Due to the nature of the C+M approach, it is at least as powerful as the C approach. Another recently introduced unconditional strategy by Lloyd [11] is that based on estimation and maximization (referred to as the E+M approach). The estimated p-value is first obtained by replacing unknown nuisance parameters in the null distribution with their maximum likelihood estimates (MLEs) using the data; the E+M p-value is then obtained by maximizing the tail probability using the estimated p-value as a test statistic. The E+M approach has been successfully applied to many important statistical and medical problems, such as testing about the difference between two independent proportions [12], [13], the Hardy-Weinberg equilibrium test [14], the difference between two incidence rates [15] and trend tests for binary endpoints [16], [17].

The rest of this article is organized as follows. In Section 2, we briefly review the existing conditional approach and consider three exact unconditional approaches. In Section 3, we compare the performance of the competing tests, studying the actual type I error rate and power of the procedures under a wide range of conditions. A real example from physical therapy is illustrated for the various testing procedures at the end of this section. Section 4 is given to discussion.

## Testing Procedures

Suppose 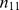 and 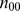 are the number of times that the conclusion from both clinicians is Yes or No, respectively. 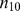 and 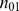 denote the number times that both clinicians do not agree with each other, 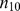 for Yes from clinician A and No from clinician B, and 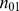 for the opposite. Let 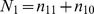 and 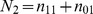 be the marginal totals for clinician A and clinician B with Yes as the diagnostic result, and 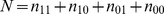 be the total sample sizes. Such data can be organized in a 2 by 2 table, such as Table 1. Let 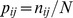 be the frequency probability, where 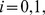 and 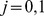. Let 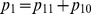 and 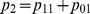 be the marginal probabilities for the first rater and the second rater, respectively, where 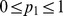 and 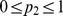. Cohen's kappa coefficient [6] is given as 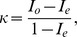
where 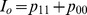 is the observed proportion of agreement, and 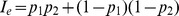 is the expected proportion of agreement on the basis of chance alone. It should be noted that weighted kappa [18] is equal to Cohen's kappa for the data in a 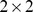 table. Landis and Koch [6] have proposed the standard for strength of agreement using the kappa coefficient, see Table 2. An alternative standard to measure the strength of agreement can be found in Martin Andres and Femia Marzo [4].

**Table 2:** Strength of agreement using the kappa coefficient.

Poor:	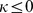
Slight:	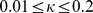
Fair:	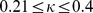
Moderate:	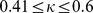
Substantial:	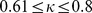
Almost perfect:	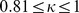

Treating the total sample size 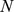 as fixed, the random vector (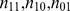) is multinomially distributed with parameter (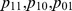), and the probability of an observed data point [19] is given as 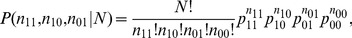
(1)where 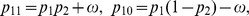 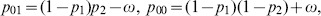 and 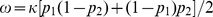.

In the problem of testing random agreement, one may be interested in the hypotheses as 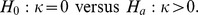



It has been pointed out by Sim and Wright [20] that a one-sided hypothesis testing problem is often considered to be appropriate when the observed agreement is equal to the agreement by chance under the null hypothesis, because a negative 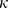 value generally does not have a meaningful practical interpretation. The interest in this article is to establish the hypothesis that the observed agreement is greater than the agreement by chance. In addition, the range of 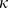 is not always from −1 to 1, it may not be appropriate to conduct two-sided hypotheses testing when the null states a zero value for the 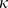 coefficient.

Under the null hypothesis, it follows that 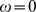 because of the relationship between 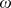 and 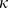 as 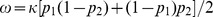. Then, 
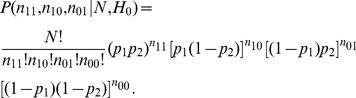
(2)


### 2.1 Conditional test

There are two nuisance parameters in the null likelihood, 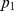 and 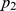 (see, Eq 2). Elimination of nuisance parameters has been studied for decades and significant progress has been achieved in this area, see [7]–[9], [11], [21]. Among them, a commonly implemented approach utilized in current commercial software for a number of problems is based on the C approach [7]. The marginal totals in the contingency table are considered to be fixed in finding the null reference distribution. This approach has been extensively investigated by Mehta et al. [22] for various classical categorical data analysis, and has been shown to be preferable to asymptotic approaches due to the guarantee of the type I error rate.

Let 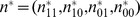 be the observed data. Given marginal totals 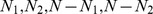, the null likelihood distribution of the C approach is constructed by enumerating all possible value of 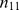, and the associated p-value is given as 
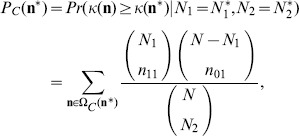
(3)where 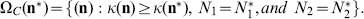 The conditional null distribution consists of a small set of unique values of the test statistic when sample sizes are small, thereby resulting in a testing procedure which performs in a conservative manner when evaluated within the unconditional framework.

### 2.2 Unconditional tests

An alternative to eliminate nuisance parameters was described by Basu [9], where the p-value is maximized over the nuisance parameter space. The associated M p-value is defined as 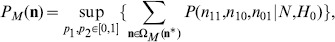
(4)where 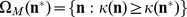 is the tail area. Traditional grid searches may be used to find the maximum of the tail probability over the region 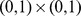. It should be noted that the choice of nuisance parameters would not affects the p-value calculation. The computational time increases exponentially with an increase in sample size. Since the ranges of parameters are limited, the function 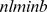 in the software R is chosen to search for the maximum with multiple initial points [23].

The Cohen's kappa 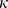 is a commonly used measure of agreement between two raters, and provides a way for the data ordering in the M approach. Boschloo [10] considered the C p-value as the ordering method in the C+M approach. Here, the C p-value is used as a test statistic, not the p value. The enumerated data is sorted by the C p-value, and the C+M p-value is then obtained by treating the C p-value as a test statistic. The corresponding tail area for the C+M p-value of the proposed test is 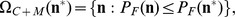
and the corresponding p-value is 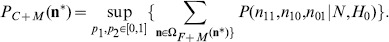
(5)


It is easy to show that the C+M approach would be at least as powerful as the C approach [10].

A simple and naive way to accommodate nuisance parameters is the plugging in method. Nuisance parameters are eliminated by replacing them with their estimated MLEs under the null. For given data, the rejection area of this estimated approach is the same as that of the M approach. The estimated p-value is given as 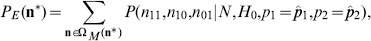
where 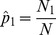 and 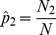 are the MLEs of 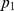 and 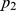, respectively.

While use of the estimated p-value does not result in an exact procedure, an exact method may be obtained by combining the estimated p-value and a maximization step [11]. The estimated p-value in this testing procedure is considered the alternative for data ordering. The corresponding tail area for the E+M p-value of the proposed test is 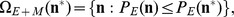
and the corresponding p-value is given as 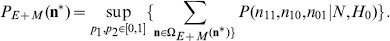
(6)


## Numerical Study

**Figure 1:**
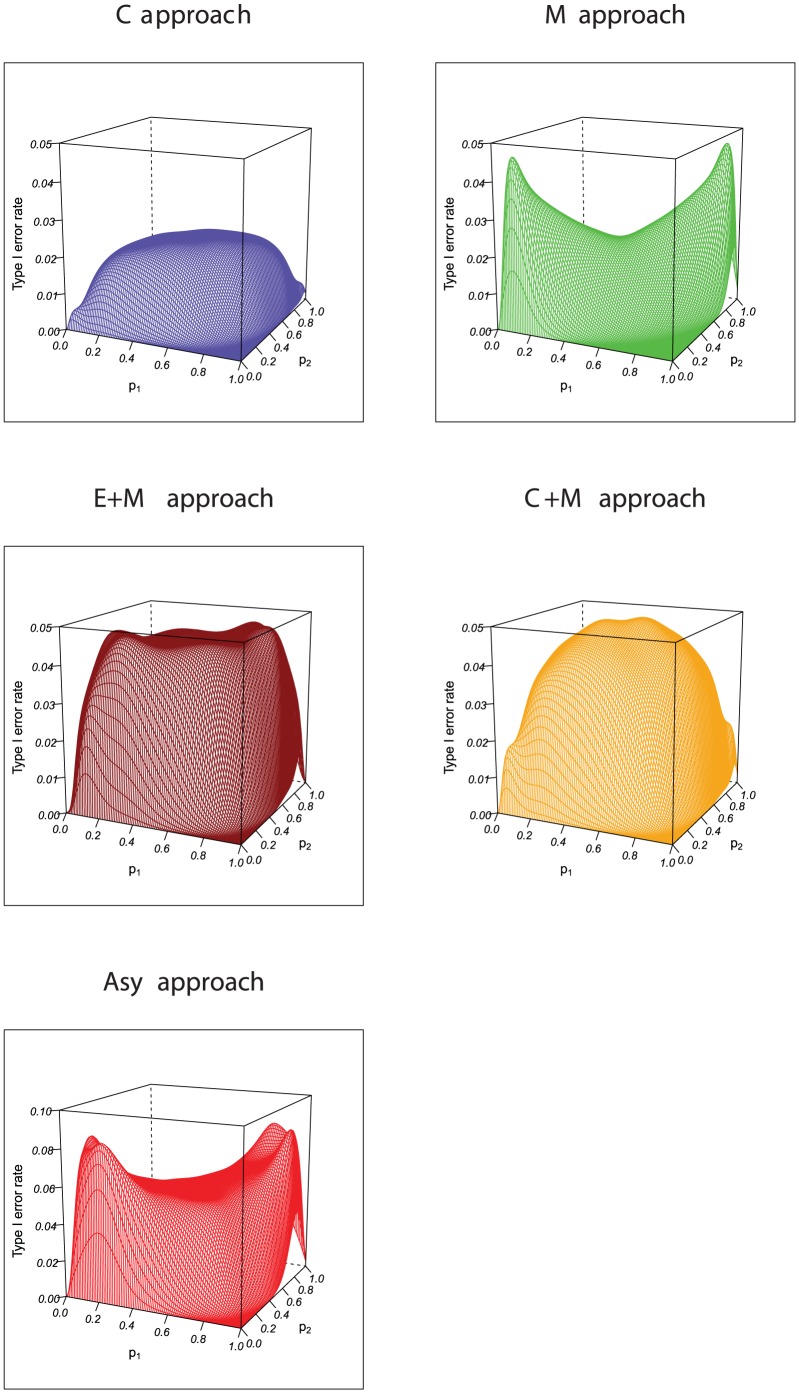
Type I rate error plots for the asymptotic, C, M, C+M, and E+M approach with N = 30.

The evaluation of the competing procedures in this note is based on enumerating all possible tables for given 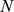, that is, there is no simulation involved. There are two nuisance parameters in the null likelihood, therefore the type I error may be expressed in a three-dimension plot. We use this plot to illustrate the unsatisfactory type I error rate for the asymptotic approach based on the standardized kappa, and the guarantee of the type I error rate for exact approaches. The type I error rate surface plots, for the five approaches with sample size 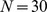 at the nominal level 0.05 are displayed in Figure 1. As seen in the figure, the majority of the points for the asymptotic approach are over 0.05. As expected, all four exact approaches respect the type I error, having the maximum of the surface less than the nominal level. It can be seen from the figure that the C approach is conservative when compared to the C+M approach and the E+M approach. The M approach is not as good as the C+M approach and the E+M approach as the surface is not as close as to the nominal level for the M approach. Table 3 shows the actual type I error rates for the asymptotic approach, the C approach, the M approach, the C+M approach, and the E+M approach at 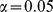 when 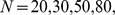 and 100. The asymptotic approach does not preserve the test size, and all other exact approaches control the type I error rate. The C+M approach and the E+M approach have actual type I error rates much closer to the nominal level than others.

**Table 3:** Actual type I error rates at 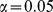.

	Testing procedure				
N	Asymptotic	C	M	C+M	E+M
20	0.0833	0.0188	0.0445	0.0462	0.0499
30	0.0837	0.0228	0.0461	0.0486	0.0474
50	0.1001	0.0295	0.0420	0.0482	0.0498
80	0.0901	0.0314	0.0436	0.0499	0.0499
100	0.0925	0.0326	0.0467	0.0499	0.0499

**Figure 2:**
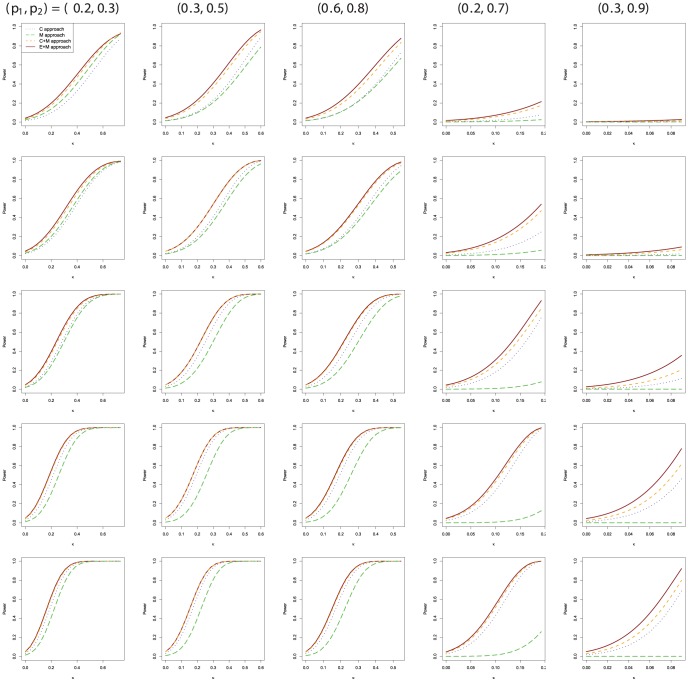
Power comparison between the four exact testing procedures for N = 20, 30, 50, 80, and 100 from row 1 to row 5, respectively.



We further compare the exact testing procedures with regards to power under a wide range of conditions. The asymptotic approach is not included in this comparison due to the unsatisfied type I error control. The power of each approach is a function of three parameters 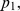 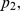 and 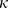. Five selected combinations of 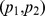 are chosen for power comparison, being 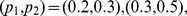 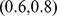, 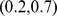, and 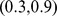. Figure 2 shows the power plots of the exact testing procedures as a function of 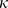 under five different pairs of 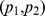 and sample sizes 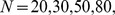 and 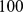. Given 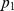 and 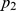, the maximum of 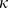 is given as

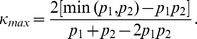



The 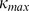 depends on the marginal probabilities, for example, 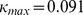 when 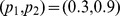. Each power plot is an increasing function of 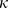. For all the cases, the M approach generally has less power than the others. Although the difference between the C approach and C+M approach (the E+M approach) becomes smaller as N increases, we still observe substantial power gain with the C+M approach and the E+M approach as compared the C approach. Although the E+M approach has more power as compared to the C+M approach in some cases, the difference between them is generally small. The plots for 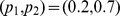, and 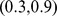 seem very different from the plots for the other three parameter configurations because the 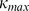 values are different from each other. When we compare the power plots for 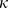 from 0 to 0.09, they have similar patterns.

### 3.1 An example

We revisit the example from Smedmark et al. [1]. The estimated prevalences (.i.e the probability of diagnosing spinal stiffness) for clinicians A and B were found to be 5% and 15%, respectively. Given the smaller sample size and evidence that the true values of the prevalence are near the boundary of the parameter space, the testing procedures offered in this manuscript may better serve as techniques to establish agreement beyond chance as compared to tests based on asymptotic null distributions. In addition to the exact procedure results, we also include the analysis of the data using the asymptotic approach for comparison sake.

We apply the following five testing procedures to the example: (1) the asymptotic approach, (2) the C approach, (3) the M approach, (4) the C+M approach, and (5) the E+M approach. The asymptotic p-value is calculated based on the asymptotic normal distribution of the standardized kappa test statistic, and the associated formula is given as 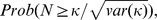
where 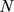 is a standard normal distribution, 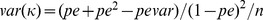, and 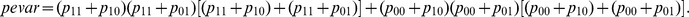 The p-values based on the asymptotic, C, M, C+M, and E+M approaches are shown in Table 4. At 0.05 significance level, the C approach and the M approach do not reject the null hypothesis since their p-values are larger than the nominal level. The asymptotic approach has a very small p-value as compared to other testing procedures. The newly considered C+M approach and the E+M approach would lead to rejection of the null hypothesis and conclude that the two clinicians agree with each other on the assessments of stiffness for the 60 patients in the study.

**Table 4:** P-values for the example assessing cervical spine stiffness.

Testing procedure				
Asymptotic	C	M	C+M	E+M
0.0051	0.0561	0.0511	0.0324	0.0205

## Conclusions

In this article we consider four exact testing procedures for testing agreement between two raters with binary outcomes. The efficient unconditional C+M and E+M approaches not only preserve the test size, but gain higher power when compared to other exact conditional or unconditional approaches. The C+M approach and the E+M approach are recommended for use in practice for small to medium sample sizes. As can be seen in Figure 2 for the power comparison, we still observe power gain for the C+M approach and E+M approach for sample sizes up to 100 as compared to other two exact approaches. The software program written in R is available from the author's website at: https://faculty.unlv.edu/gshan/Agreement.r.

In this note we focused our attention on the one-sided problem, since scientific interest is often in terms of establishing agreement beyond chance, rather than establishing agreement is more or less than that expected by coincidence. If the two-side alternative is of interest, a similar exact testing approach may be taken based on statistics such as 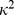 where large values would denote evidence that the true agreement is different than that of chance. In addition, procedures based on non-zero null values of the kappa coefficient may be pursued via the unconditional approach where the null value is set at constant representing a minimally acceptable threshold for agreement, ex. 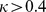. Such test statistics for use in this problem may have simple forms such as 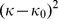. Furthermore, through inversion of such test, an exact confidence interval may be obtained which is a subject of future research.

There are many discussions about the Cohen's kappa test statistic [24], [25], and some test statistics have been proposed to deal with the imbalance in the tables' marginal totals, such as the kappa max [26], the delta model [4]. Applying efficient exact testing procedures for testing agreement between two raters with 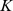 nominal outcomes [27] is currently underway.
